# A study on vaginitis among pregnant and non-pregnant females in Alexandria, Egypt: An unexpected high rate of mixed vaginal infection

**DOI:** 10.3934/microbiol.2022014

**Published:** 2022-05-05

**Authors:** Sherine Mohamed Shawaky, Mariam Majed Ali Al Shammari, Manal Shafik Sewelliam, Abeer Abd El Rahim Ghazal, Ahmed Noby Amer

**Affiliations:** 1 Microbiology Department, Medical Research Institute, Alexandria University, Egypt; 2 Department of Obstetrics and Gynecology, Faculty of Medicine, Alexandria University, Egypt; 3 Microbiology and Immunology Department, Faculty of Pharmacy, Pharos University, Alexandria, Egypt

**Keywords:** bacterial vaginosis, Gardnerella vaginalis, Amsel's criteria, Nugent's scores, candidiasis, *Trichomonas vaginalis*, mixed vaginal infections

## Abstract

**Background:**

Many infectious and noninfectious triggers lead to inflammation of the vagina.

**Aim:**

We investigated the prevalence of causative vaginitis microorganisms in 516 pregnant and nonpregnant female volunteers. Vaginal samples were examined microscopically, cultured and tested for different pathogens.

**Results:**

Of the participants, 310 (60.1%) were pregnant, whereas 206 (39.9%) were nonpregnant. Using Amsel's criteria and Nugent's scores, bacterial vaginosis (BV) was diagnosed in 59.1%, and the prevalence of vulvovaginal candidiasis (VVC) was 50.2% in the population. *Candida* infections were significantly higher in nonpregnant females (p value ≤ 0.01), and 24% of females had mixed infections. The most common mixed infection was BV and *Candida* spp., detected in 21% of the cases.

**Conclusions:**

Bacterial vaginosis is the most common cause of vaginitis. We observed that 24% of females experienced mixed infections, and *Candida albicans* was the most common fungal species causing VVC. *Trichomonas vaginalis* prevalence was underestimated using wet mounts.

## 1. Introduction

Most females experience vaginitis at least once in their lifetime [1]. As one of the most commonly reported vaginal infections, the condition accounts for more than 10% of cases in health care clinics [2]. Vaginitis generally refers to inflammation of the vaginal wall, with several infectious and noninfectious triggers responsible for this inflammation. Bacterial vaginosis (BV), vulvovaginal candidiasis (VVC) and trichomonal vaginitis are the most common vaginal infections [3]. *Lactobacillus* spp. constitute over 95% of vaginal bacteria and are the predominant microorganisms [4]. They are believed to protect the vagina against infection, in part by maintaining an acidic pH and ensuring a hydrogen peroxide environment in the vaginal atmosphere [5]. BV is the most common cause of abnormal vaginal discharge, is mediated by disturbances in vaginal microbiota and may be temporary or permanent [6]. Also, *Candida* species, in particular *Candida albicans*, are part of the normal vaginal microbiota; however, its presence is often asymptomatic. Thus, a VVC diagnosis requires the comprehensive laboratory investigation of all symptoms [7].

Vaginitis is very common, especially in developing countries such as Egypt [8]. Also, vaginal infection during pregnancy is associated with a high incidence of preterm labor and neonatal ICU admission [9].

In this study, we investigated the prevalence of causative vaginitis microorganisms using clinical methods, conventional laboratory methods and molecular applications, in both pregnant and nonpregnant females. Also, the study assessed the main risk factors associated with vaginitis.

## 2. Materials and methods

The study was conducted between September 2019 and March 2020. A total of 516 vaginal swabs were collected from female patients admitted to El-Shateby University Hospital. All patients were experiencing vaginitis with one or more of the following symptoms: change in color and odor of vaginal discharge, pain during intercourse, vaginal itching and inflammation and dysuria.

A full medical history was taken from each patient; this included the evaluation of several risk factors such as diabetes mellitus (DM), hypertension, anemia, and frequency of intercourse.

### 2.1. Direct examination

Besides physical examination, direct examinations included vaginal pH tests using pH paper and the whiff test to detect BV; a fish odor after treating samples with 10% potassium hydroxide (KOH) indicates positive results [10].

### 2.2. Sample collection, microscopy and BV evaluation

Three swab/discharge samples were collected from patients and immediately transported to the laboratory. On a slide, vaginal discharge was examined for *Candida* by mixing samples with 10% KOH and examining under a microscope, and candidiasis was diagnosed by hyphae and budding yeast cells [11]. On another slide, vaginal discharge was mixed with saline and used to detect *Trichomonas vaginalis*, a motile flagellated pear-shaped organism. Also, this film was used to evaluate the presence of clue cells, which are vaginal epithelial cells with adherent coccobacilli. An increased number of round parabasal cells was considered a sign of atrophic vaginitis [12],[13]. A third slide was used for Gram-stain; prepared mounts were examined and quantified for *Lactobacillus*, *Bacteroides/Gardnerella*, and *Mobiluncus* for calculation of Nugent's score [14]. A rapid diagnostic test was conducted using Liofilchem TM S. l R strips to determine *Gardnerella* spp. in samples.

BV was diagnosed using the following two methods: (a) Amsel's criteria established a positive BV diagnosis if three of the following four clinical signs were present: 1) thin, gray and homogenous discharge; 2) a vaginal secretion of pH > 4.5; 3) a positive whiff test; and 4) the presence of clue cells on wet mounts [15],[16]. (b) Nugent's scores [17] were generated using a semiquantitative evaluation method to estimate the relative prevalence of *Lactobacilli*, *G. vaginalis/Bacteroides*, and *Mobiluncus* in Gram-stained vaginal wet mounts: 0–3 = normal, 4–6 = intermediate, and 7–10 = BV.

### 2.3. Molecular identification

Samples showing no pathogenic growth upon culturing, a negative microscopy result and a *G. vaginalis* rapid test, were investigated using real-time PCR. Immediately after collection, a vaginal swab was added to a tube containing 500 µL sterile-buffered saline and mixed thoroughly. If real time PCR was needed, DNA was extracted using a DNA extraction kit (PREP-GS) (www.dna-technology.ru). The Femoflor Screen real-time PCR detection kit was used to determine the presence and relative abundance of vaginal normal flora, opportunistic flora and pathogens using multiplex quantitative real-time PCR. The kit is designed to detect and quantitively determine a wide range of etiologically significant opportunistic aerobic organisms and anaerobic organisms; also, it can detect *Mycoplasma* spp. and *Candida* spp. Moreover, it can detect genital pathogens such as *Chlamydia trachomatis*, *Neisseria gonorrhoeae*, *T. vaginalis*, and viral pathogens (Cytomegalovirus, herpes simplex viruses 1 and 2). The test was prepared according to the manufacturer's guidelines. Positive and negative controls were supplied with the kit. The negative control was subjected to the whole DNA extraction procedure. Both controls were added to each strip in separate tubes.

Data were analyzed using SPSS (ver. 20, Chicago, IL, USA). Pearson's chi square test was used for association between categorical variables and expressed by p-value; the level of significance was 0.05, below which the results were considered to be statistically significant.

Approval of this study was obtained from the Ethical Committee at the Medical Research Institute, Alexandria University, approval number E\C. S/N. T15/2019. An informed consent was obtained from all participants before sampling. The study was conducted in accordance with the standards set forth in the Declaration of Helsinki principles of 1975, as revised in 2013 (http://ethics.iit.edu/ecodes/node/3931).

## 3. Results

In this study, 310 (60.1%) females were pregnant and 206 (39.9%) were not. The majority were in their 20s and 30s (mean age: 29.92 ± 8 years). Vaginal discharge was the most common symptom (81.7%), followed by itching (58.1%) and dysuria (14.7%), whereas 12.4% complained of painful sexual intercourse. Itching was the most common symptom among nonpregnant females, whereas vaginal discharge was the common symptom among pregnant females. We observed no significant differences in symptom distribution between pregnant and nonpregnant females, except for painful sexual intercourse, which was significantly higher in nonpregnant females (Table 1).

Among the reported risk factors (Table 1), diabetes mellitus (DM) was the most common (18.2%), followed by anemia (15.3%). Hypertension was significantly higher among pregnant females. However, in 47.5% of females, no other risk factors were identified (Table 1).

**Table 1.** Distribution of age, risk factors, and symptoms among the study groups.

	Pregnant	Nonpregnant	Total
Total no.	310 (60.1%)	206 (39.9%)	516 (100%)
Mean age	28.71 ± 7.23	31.75 ± 8.79	29.92 ± 8
Signs and symptoms
Discharge	250 (80.6%)	172 (83.4%)	422 (81.7%)
Itching	177 (57.1%)	123 (59.7%)	300 (58.1%)
Dysuria	42 (13.5%)	34 (16.5%)	76 (14.7%)
Painful sexual intercourse	31 (10%)*	33 (16%)*	64 (12.4%)
Inflammation & redness	18 (5.8%)	12 (5.8%)	30 (5.8%)
Risk factors	Reported in 159 (51.3%)	Reported in 112 (54.4%)	Reported in 271 (52.5%)
Diabetes mellitus	52 (16.8%)	42 (20.4)	94 (18.2%)
Hypertension	13 (4.2%)**	5 (2.4%)**	18 (3.4%)
Frequent intercourse	44 (14.2%)	25 (12.1%)	69 (13.4%)
Anemia	44 (14.2%)	35 (17%)	79 (15.3%)

*Note: For χ^2^ (chi-squared) test: * significant at p ≤ 0.05; ** highly significant at p ≤ 0.01.

All Amsel criteria were identified in only 31.4% of females. The most reported single criteria were homogenous discharge (67.4%), positive whiff test (54.7%) and the presence of clue cells (50.2%). Clue cell numbers were statistically higher in the nonpregnant group (60.7%). The presence of all three criteria together was significantly higher in nonpregnant than in pregnant females.

In terms of Nugent's scores, the majority of cases (44.2%) scored 7–10, which indicated BV, whereas 28.7% scored 4–6 (intermediate), and 27.1% scored 1–3 (normal or negative for BV) (Table 2).

**Table 2.** Comparison of Amsel's criteria and the calculated Nugent's score among pregnant and non-pregnant groups.

	Pregnant	Nonpregnant	Total
Amsel's criteria			
Clue cells	134 (43.2%)**	125 (60.7%)**	259 (50.2%)**
Whiff test	161 (51.9%)	121 (58.7%)	282 (54.7%)
Homogenous discharge	201 (64.8%)	147 (71.4%)	348 (67.4%)
All criteria present	74 (23.9%)**	88 (42.7%)**	162 (31.4%)**

Nugent Score			
Normal (1–3)	75 (24.2%)	65 (31.6%)	140 (27.1%)
Intermediate (4–6)	97 (31.3%)	51 (24.8%)	148 (28.7%)
Bacterial vaginosis (7–10)	138 (44.5%)	90 (43.7%)	228 (44.2%)

*Note: For χ^2^ (chi-squared) test: * significant at p ≤ 0.05; ** highly significant at p ≤ 0.01.

We identified a week association between Amsel's criteria and Nugent's scores for BV (p < 0.01). We observed that 211 (40.9%) females were negative for Amsel's criteria and Nugent's score when compared with 85 (16.5%) positive females. However, 77 females (14.9%) were positive for Amsel's criteria while Nugent's scores were negative. However, 143 (27.7%) females were negative for Amsel's criteria while Nugent's scores were positive.

Using Gram-stain, culturing, Amsel's criteria, Nugent's scores and *G. vaginalis* rapid tests, BV was diagnosed in 59.1% (305/516) of females. Using rapid testing, *Gardnerella* spp. represented 65.9% (201/305) of BV cases, with a total prevalence of 39% among females. *Candida* was identified in 50.2% of females (259/516), with GBS in 5.6% and *T. vaginalis* in two females (Table 3).

**Table 3.** Causative agent of vaginitis detected by conventional methods and real time PCR among pregnant and nonpregnant women.

Diagnosis	Pregnant	Nonpregnant	Total
Identified by conventional methods
Total bacterial vaginosis	211 (68.1%) *	94 (45.6%)*	305 (59.1%)
*Gardnerella vaginalis*	139 (44.8) **	62 (30.1) **	201 (39%)
*Candida* spp.	141 (45.5%) **	118 (57.3%) **	259 (50.2%)
GBS (Group B Streptococci)	17 (5.5%)	12 (5.8%)	29 (5.6%)
*Trichomonas vaginalis*	1 (0.3%)	1 (0.5%)	2 (0.4%)

Identified by PCR (45 cases)			
*Lactobacilli*	10 (3.2%)	13 (6.3%)	23 (4.5%)
*Lactobacilli, G. vaginalis* / *Prevotella* sp.	3 (0.96%)	3 (1.5%)	6 (1.2%)
*Gardnerella vaginalis* / *Prevotella* sp.	4 (1.3%)	3 (1.5%)	7 (1.4%)
*Ureaplasma*	1 (0.32%)	2 (0.97%)	3 (0.58%)
*Mycoplasma genitalium*	0	1 (0.49%)	1 (0.19%)
*Mycoplasma hominis*	1 (0.32%)	1 (0.49%)	2 (0.38%)
*Trichomonas vaginalis*	2 (0.65%)	1 (0.49%)	3 (0.58%)

*Note: For χ^2^ (chi-squared) test: * significant at p ≤ 0.05; ** highly significant at p ≤ 0.01.

We observed that 24% of females (124/516) had mixed infections. Mixed *Candida* species infections with BV were identified in 108 cases (21%). Also, GBS coinfection with *Candida* species was identified in 10 cases (1.93%), and that with *T. vaginalis* was identified in two cases. Colonization with both GBS and BV was identified in four cases.

The *Candida* and BV isolation rate was significantly higher in nonpregnant females, whereas *Gardnerella* detection rates were statistically higher in pregnant females. In terms of other vaginitis causes, GBS and *T. vaginalis* were detected in 17 (5.5%) and one (0.3%) pregnant female(s), respectively, when compared with 12 (5.8%) and one (0.5%) nonpregnant female(s), respectively (Table 3).

Forty-five cases uncharacterized by conventional methods were identified using real-time PCR. *G. vaginalis Prevotella* were detected in 13 cases, with or without *Lactobacilli*. *Ureaplasma*, *Mycoplasma genitalium* and *Mycoplasma hominis* were detected in three (0.58%), one (0.19%) and two (0.38%) cases, respectively. Three more *T. vaginalis* cases were also identified via real-time PCR. Thus, the overall prevalence of *T. vaginalis* using conventional methods and real time PCR was 5/516 (0.98%). *Neisseria, Chlamydia and viral pathogens were not detected*.

We identified 259 *Candida* isolates at the species level using *Candida* chromogenic agar (*Candida albicans*, *Candida glabrata*, *Candida tropicalis* and *Candida kruesi*. Two species were identified using VITEK 2 technology (*Candida dubliniensis* and *Candida guilliermondii*). *C. albicans* was a major causative agent of vaginal candidiasis (194/259, 74.9%). Non-*C*. *albicans* isolates were also detected in 65/259 (25.1%) cases: *C. tropicalis* was the most common non-*C*. *albicans* type (31/259, 11.9%), followed by *C. glabrata* (21/259, 8.1%) and *C. kruesi* (11/259, 4.2%).

In terms of antifungal agent sensitivity, all *Candida* species were sensitive to ketoconazole, miconazole, micafungin, nystatin, clotrimazole, and caspofungin. For fluconazole, all *C. kruesi* isolates were intrinsically resistant, whereas six (3.1%) *C. albicans* and six (19.4%) *C. tropicalis* isolates were resistant. Also, two *C. albicans* (1.14%) and one *C. tropicalis* (3.2%) isolate were resistant to voriconazole. Two *C. albicans* isolates were resistant to amphotericin B.

## 4. Discussion

Vaginitis is defined as vaginal inflammation and is typically characterized by several signs and symptoms, including vaginal discharge, vulvar itching, vaginal erythema and dysuria [3].

The majority of females in this study were in their 20s and 30s. Pregnant females were more common (60%). Several other studies showed similar age group prevalence. Mahakal et al. [21] found that among 272 females included in their study, the commonest age-group with vaginal discharge was 20–40 years (83%), among whom only 21% of females were pregnant. Similar results were reported in an Indonesian descriptive study [22] comprising 492 women, where the average age was 30.9 ± 0.27 years.

Vaginal discharge was the most common symptom (81.7%) presented, followed by itching (58.1%). This was contrary to Mahakal *et al*. [21], who reported that 83% of their patients presented with pruritus, and 46% and 44% of them complained of dysuria and dyspareunia, respectively.

We observed no significant differences in symptom distribution between pregnant and nonpregnant females. However, painful sexual intercourse was significantly higher in the nonpregnant group and was putatively explained by less frequent sexual intercourse when compared with pregnant females.

In our study, no risk factors were identified in 47.6%. Among the reported risk factors, DM was the most common (18.2%), followed by frequent sexual intercourse (13.4%). Diabetes mellitus predisposes to Candidiasis, as well as bacterial infections. Vulvovaginal candidiasis (VVC) has been proposed by many investigators in diabetics to be more frequent [23],[24]. Furthermore, chronic recurring VVC may be a marker of diabetes [25].

European guidelines recommend a BV diagnosis may be based on clinical symptoms and signs when supported by additional experimental findings. Thus, Amsel's clinical criteria, Nugent's scores or culture-based techniques can be used [26].

We diagnosed BV in 305/516 (59.1%) females using either Nugent's scores or Amsel's criteria. A weak agreement was identified between these indices for BV. Several studies have reported that Amsel's criteria are as good as Nugent's scores for diagnosing BV; both are simple, easy, cost effective and reliable methods [27]. However, by applying molecular techniques, we also diagnosed a further 13 additional BV cases. As both Nugent's scores and Amsel's criteria have inherent limitations, DNA-based assays targeting BV are reproducible and may be used to diagnose difficult cases [28]. A lower BV prevalence was reported in an Ethiopian study, in which BV was diagnosed using Nugent's score in 20.1% of the patients [29]. The prevalence in other African countries was reported to be also lower; in a meta-analysis, the prevalence of BV in sub-Saharan Africa was 42.1% [30]. The higher prevalence in our study could be explained by the usage of more than one technique for diagnosis of BV. Also, there are differences in the prevalent microbial populations between countries [31].

In this study, 50.2% of females were diagnosed with VVC. *Candida* infections were significantly higher in nonpregnant females and may have been exacerbated by pregnancy, high oral estrogen contraception use, antibiotics and health conditions such as DM, which was the highest reported predisposing factor among pregnant and nonpregnant females in our study. VVC also appears to be more frequent in diabetics [32],[33]. Uncommon, non-*C. albicans* strains were also identified using VITEK 2 technology: *C. dubliniensis* (1/65, 1.53%) and *C. guilliermondii* (1/65, 1.53%).

In terms of *Candida* distribution, our data agreed with Hassan *et al*. (2017) [27]. We identified six *Candida* species: *C. albicans* was the predominant strain (62.9%), followed by *Candida lusitaniae* (13.7%), *C. krusei* (10.7%), *C. glabrata* (6.3%), *C. tropicalis*, and *Candida parapsilosis* [34]. Previously, in an Argentinian study, 55 *Candida* isolates or other yeasts were identified in patients with VVC; *C. albicans* was the predominant species (85%), followed by *C. dubliniensis* and *C. glabrata* [35].

Identifying yeast at the species level is vital for choosing appropriate antifungal treatments. *C. albicans* is the main pathogen seen in VVC; however, other *Candida* species, as presented in our study, may not share the same susceptibility profile with *C. albicans*. This could lead to recurrent VVC, which is distinguished from persistent infection by symptom-free intervals [36]. The reported *Candida* resistance to antifungal drugs could be attributed to empirical long-term treatment without identifying species and susceptibility.

We identified *T. vaginalis* in five females (0.96%), two using wet mounts and three using real-time PCR. Similar findings were identified in a cross-sectional study [37] in reproductive-age females. Using microscopy, these authors reported *T. vaginalis* as the least frequent cause of vaginitis, with a detection rate of < 1.0% in their cohort. This low prevalence contrasted with a Vietnamese study [38] where 249 symptomatic and 534 asymptomatic females had an overall trichomoniasis prevalence of 6.6% by microscopy.

We identified GBS in 5.6% of females; however, there was no significant difference in the prevalence between pregnant and nonpregnant females. GBS are part of the typical recto-vaginal flora and are transiently identified in asymptomatic females [39]–[42]. In another Egyptian study, a higher GBS prevalence (25.3%) was identified using vaginal swabs cultured on ISLAM'S media [43]. The sensitivity of the ISLAM media used may explain the difference in the prevalence.

The detection of genital *Mycoplasma* in females is important and is augmented by the high rate of vertical transmission and associations with neonatal morbidity and mortality [44]. *Mycoplasma* culturing is costly and requires specialized media and expertise. Infectious agents may be detected in 8 h using nucleic acid amplification techniques. In our study, we used multiplex real-time PCR to characterize vaginitis etiology in females where no definitive causes were identified using conventional methods. Using real time PCR, three *Ureaplasma* species, *M*. *genitalium* (1) and *M. hominis* (2) were identified in 45 females.

However, using culturing techniques, discrepant prevalence rates were reported in Australia [38]. Out of 175 cervical swabs cultured from Australian females, with and without cervicitis, 16% tested positive for *M. hominis* and 68% for *Ureaplasma* species [45]. This difference in prevalence may be explained by different sexual behaviors and restricted sexual activities in Egypt when compared with Western countries. Additionally, only 45/516 samples with no obvious cause for vaginitis were tested in our study.

We detected no viral pathogens. This result may be explained by several factors: Only one sample per patient was tested, and viral loads below detection limits may have been missed. We used commercially available specimen collection swabs and transportation media, which may not have provided optimum conditions for viral nucleic acid recovery. Moreover, the majority of the females included in this study did not present with genital ulcers, potentially explaining the absence of vaginal viral infections.

Alarmingly, we identified mixed vaginal infections in 24% of females, suggesting that nearly one in four may be diagnosed and treated for one causative pathogen, with the other(s) left undiagnosed, thereby prolonging patient suffering. The most commonly observed mixed infection was VVC with BV (21%). Although vaginal infections are extremely common, little information is available on mixed infection prevalence, especially VVC and BV. The co-incidence of microscopy-defined VVC and BV varies considerably and ranges from 3% to 27% [46],[47]. In spite of this high incidence, these infections remain poorly studied. Thus, improved diagnostic procedures are recommended to facilitate mixed infection diagnoses and provide appropriate and timely therapeutic options [48].

## 5. Conclusions

BV is the most common cause of vaginitis. *C. albicans* is the most common fungal species causing VVC, with a low rate of antifungal resistance. Species identification and susceptibility testing for VVC patients is recommended, especially for recurrent cases. *T. vaginalis* may be underestimated using conventional diagnostic methods. Mixed vaginal infections remain underestimated in clinical practice. It is recommended that diagnostic methods be improved to capture potential mixed infection risks and bridge the therapeutic gap using combination therapy.
